# Effect of Intrinsic Point Defect on the Magnetic Properties of ZnO Nanowire

**DOI:** 10.1155/2013/541496

**Published:** 2013-12-12

**Authors:** Jiangni Yun, Zhiyong Zhang, Tieen Yin

**Affiliations:** School of Information Science and Technology, Northwest University, Xi'an 710127, China

## Abstract

The effect of intrinsic point defect on the magnetic properties of ZnO nanowire is investigated by the first-principles calculation based on the density functional theory (DFT). The calculated results reveal that the pure ZnO nanowire without intrinsic point defect is nonmagnetic and ZnO nanowire with V_O_, Zn_i_, O_i_, O_Zn_, or Zn_O_ point defect also is nonmagnetic. However, a strong spin splitting phenomenon is observed in ZnO nanowire with V_Zn_ defect sitting on the surface site. The Mulliken population analysis reveals that the oxygen atoms which are close to the V_Zn_ defect do major contribution to the magnetic moment. Partial density states calculation further suggests that the appearance of the half-metallic ferromagnetism in ZnO nanorod with V_Zn_ originates from the hybridization of the O2p states with Zn 3d states.

## 1. Introduction

Recently diluted magnetic semiconductors (DMSs) have attracted much attention due to their potential application in novel spintronic devices. Among these DMSs, low dimensional ZnO-based DMSs have been found to be promising for nanometer scale optomagnetics devices, optoelectronics devices, and biotechnology [1–15]. Many investigations have been reported regarding the intrinsic ferromagnetism in undoped one-dimensional ZnO nanomaterials [9–15]. However, these reports demonstrate controversial results. For example, Xing et al. [9] have reported that the room temperature ferromagnetism (RTFM) in undoped ZnO nanowire can be attributed to the large population of Vo, since they can initiate defect-related hybridization at the Fermi level and establish a long-range ferromagnetic ordering. Ghosh et al. [10] have found that V_Zn_ was responsible for the ferromagnetic behaviour in pure ZnO nanowire. Until now, the exact mechanism of intrinsic magnetism in undoped ZnO nanowire is still controversial. On the other hand, to reveal the origin of intrinsic magnetism in undoped ZnO, many theoretical calculations have been performed to study the roles of intrinsic point defects in ZnO [16–23]. These calculation results have made important contributions to the understanding of the roles of defects in ZnO and enriched the underlying knowledge of ZnO significantly. However, most of these calculations focused on the formation energies and transition levels [17, 18, 20, 23], which could not be detected directly in experiments. To date, the comprehensive investigation about the effect of intrinsic point defect on the magnetic properties of ZnO nanowire is lacking.

In this paper, we perform the first-principles calculation based on the density functional theory (DFT) to investigate the effect of intrinsic point defect on the magnetic properties of ZnO nanowire. The method we use offers the advantage of cost-efficient alternative to conventional ab initio methods in quantum chemistry. It gives results of a quality comparable to or even better than MP2 for a cost that is on the same order as Hartree-Fock. A further advantage of DFT is that it can be significantly more efficient than even traditional SCF theory. Also the robust electron ensemble DFT approach can be used for systems with partial occupancies.

From the results obtained from this paper, a better understanding of the mechanism of intrinsic magnetic properties in undoped ZnO nanowire with sufficient details can be estimated, and some helpful instructions can be provided for the growth of low dimensional ZnO-based DMSs. These results also imply a promising way to synthesize undoped ZnO nanowire with ferromagnetism and shed light on the fabrication of ZnO-based nanometer scale magnetic devices.

## 2. Computational Details

**Figure 1:**
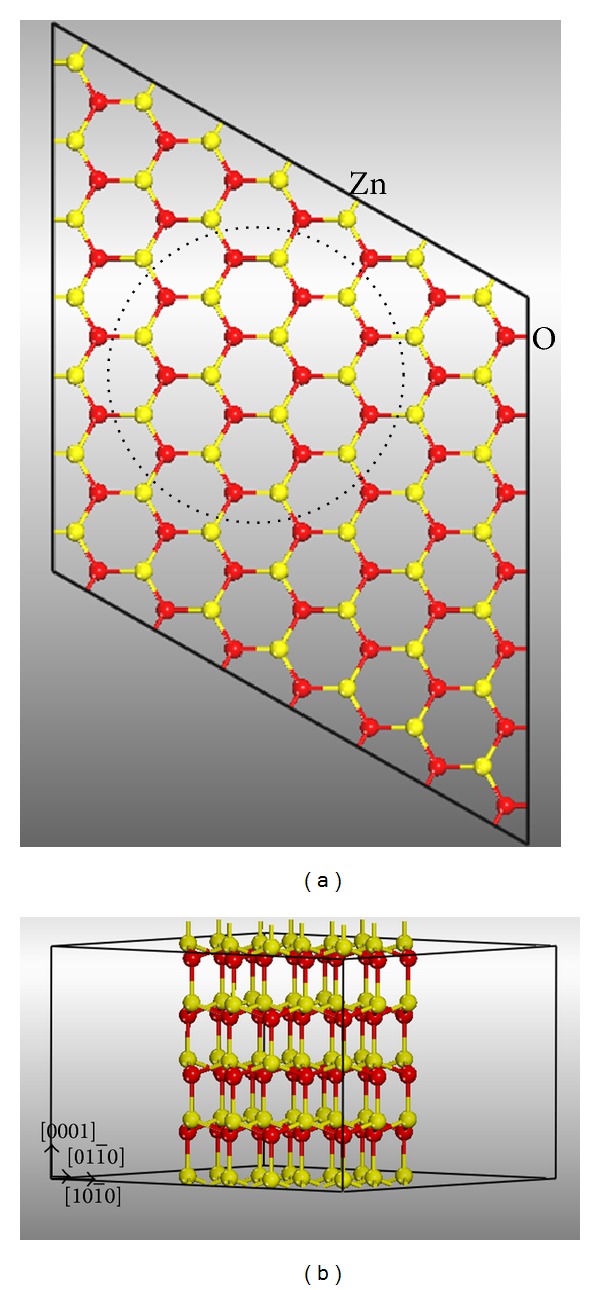
(a) Top view of a 7 × 7 × 2 ZnO supercell with wurtzite structure, (b) Zn_48_O_48_ supercell which yields a nanowire along [0001] direction.

**Figure 2:**
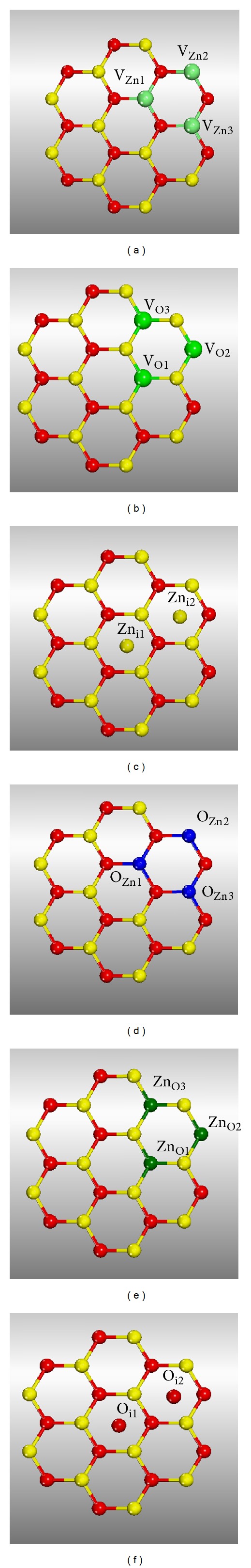
The configurations of ZnO nanowire (a) with V_Zn_, (b) with V_O_, (c) with Zn_i_, (d) with O_Zn_, (e) with Zn_O_, and (f) with O_i_ defect.

As shown in Figure 1, the ZnO nanowire is generated from a 7 × 7 × 2 supercell of bulk wurtzite ZnO along the [0001] direction. It has a diameter of 1.0 nm, corresponding to 96 atoms per unit cell. A vacuum region of about 16 Å is set to avoid the interaction of the nanowire with its image. For the ZnO nanowire with V_Zn_, V_O_, O_Zn_, or Zn_O_ defect, there are three inequivalent defect positions, which are denoted by V_Zn1_–V_Zn3_, V_O1_–V_O3_, O_Zn1_–O_Zn3_, and Zn_O1_–Zn_O3_ as shown in Figures 2(a), 2(b), 2(d), and 2(e), respectively, whereas, there are two inequivalent Zn_i_ and O_i_ defect positions, which are denoted by Zn_i1_-Zn_i2_ and O_i1_-O_i2_ as shown in Figures 2(c) and 2(f), respectively.

All the calculations are performed by using the CASTEP software package [24]. The interaction between nuclei and electrons is approximated with Vanderbilt ultrasoft pseudopotential [25] and the Perdew and Wang 91 parametrization [26] is taken as the exchange-correlation potential in the generalized gradient approximation (GGA). Plane wave basis with kinetic energy cutoff of 420 eV is used to represent wave functions. The Brillouin Zone integration is approximated using the special *k*-points sampling scheme of Monkhorst-Pack [27] and 1 × 1 × 16  *k*-points grid is used. In order to obtain a stable structure, full relaxation is performed by using the BFGS algorithm [28] to minimize energy with respect to atomic position. The spin-polarized calculations are adopted to properly describe the electronic structure and magnetic properties of the constructed ZnO nanowire models. Each calculation is considered converged when the maximum root-mean-square convergent tolerance is less than 1 × 10^−6^ eV/atom.

With the settings described earlier, we have performed test calculations for bulk ZnO with wurtzite structure to verify the accuracy of the computational method. The obtained geometry optimization crystallographic parameters of wurtzite ZnO were*a* = 3.248 Å, *c* = 5.206 Å, and *c*/*a* = 1.603, which are in good agreement with the experimental values [29], *a* = 3.250 Å, *c* = 5.207 Å, and *c*/*a* = 1.602. We also tested the total energies and electronic structures of the considered defects model and the results indicate that the 96-atom supercell is sufficient for the present calculations.

## 3. Results and Discussion

### 3.1. Structural Stability

As shown in Figure 2, there are inequivalent defect positions in ZnO nanowire with intrinsic point defect. To determine the stable configurations for each intrinsic point defect, the relative energy Δ*ε* is calculated by comparing the total energy values of different configurations, with the ground state energy taken as the reference value. From the calculated relative energy Δ*ε* listed in Table 1, it is evident that the total energy of V_Zn_ at V_Zn2_ site is about 0.12 and 0.04 eV smaller than that at the V_Zn1_ and V_Zn3_ sites, respectively, and thus, the V_Zn2_ is the most stable site for V_Zn_ defect. Similar to V_Zn_, the V_O_, O_Zn_, and Zn_O_ the also prefered at the V_O2_, O_Zn2_, and Zn_O2_ sites, respectively. While for the Zn_i_ and O_i_ defect, they are prefered at the Zn_i1_ and O_i1_ sites, respectively. Therefore, the magnetic properties of ZnO nanowire with intrinsic point defects are calculated based on the V_Zn2_, V_O2_, O_Zn2_, Zn_O2_, Zn_i1_, and O_i1_ configurations. On the other hand, the V_Zn2_ and V_Zn3_ configurations are nearly degenerate in total energy, and the total energy of them is smaller than that of the V_Zn1_ configuration. This clearly suggests that the V_Zn_ defect shows selectivity of site occupancy and prefers sitting on the surface site.

### 3.2. Magnetic Properties

**Figure 3:**
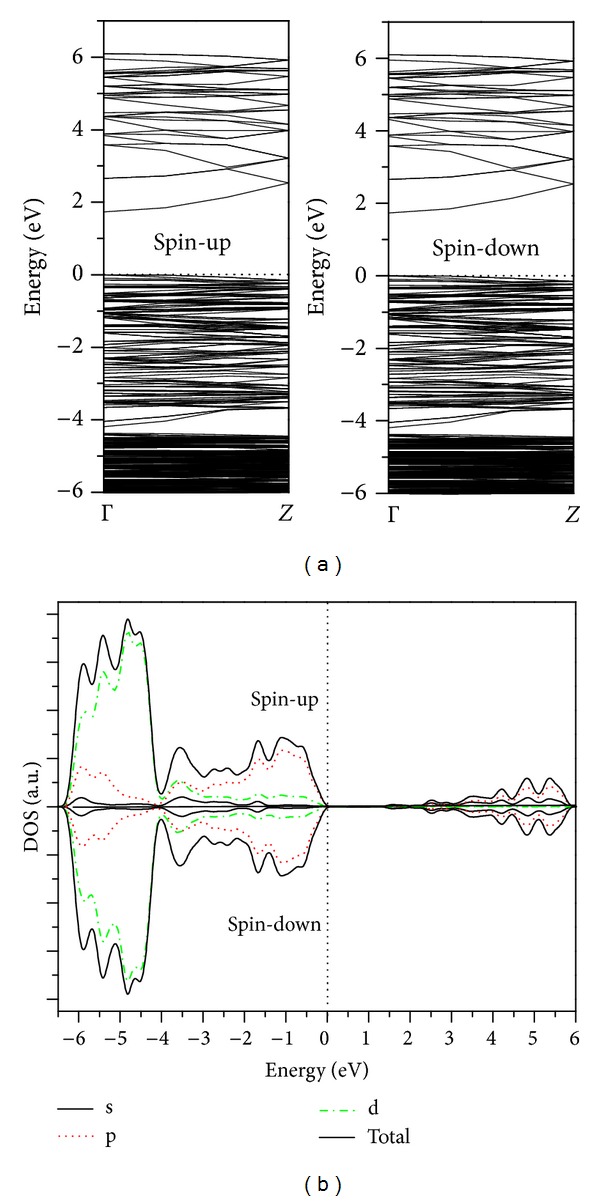
Calculated BS, total DOS, and PDOS of the pure ZnO nanowire. The Fermi level is set to zero on the energy scale, which will be adopted below unless otherwise stated.

In this part, the electronic structures of ZnO nanowire without and with different intrinsic point defect will be discussed and compared with each other. Four indicators will be used to reveal the effect of intrinsic point defect on the magnetic properties of ZnO nanowire, which are the band structure (BS), total density of states (DOS), partial density of states (PDOS), and Mulliken population analysis. Each of these tools can demonstrate some aspects of structure features. For comparison, the BS, total DOS, and PDOS of ZnO nanowire without intrinsic point defect are calculated first and the results are shown in Figure 3.

As shown in Figure 3, the spin-up and spin-down DOSs of pure ZnO nanowire are completely symmetrical, indicating that the pure ZnO nanowire is nonmagnetic. Moreover, the ZnO nanowire has a direct band gap at the relatively high dispersion along the Γ(0, 0, 0) → Z(0, 0, 0.5) direction. The direct gap of 1.86 eV is in good agreement with that of the first-principles calculation by Yang et al. [30].

**Figure 4:**
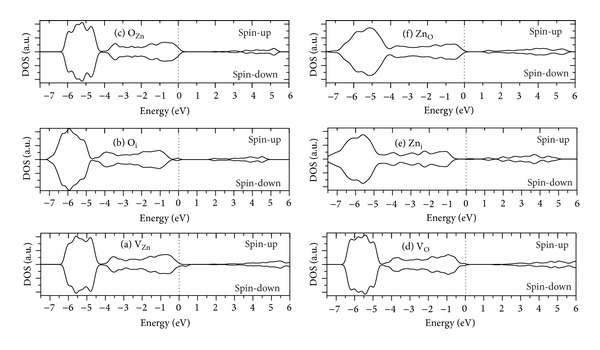
Calculated total DOS for ZnO nanowire with different intrinsic point defects.

The total DOSs for spin-up and spin-down electrons of the ZnO nanowire with V_Zn_, V_O_, Zn_i_, O_i_, O_Zn_ or Zn_O_ point defect are plotted in Figure 4. Similar to the pure ZnO nanowire, the ZnO nanowire with V_O_, Zn_i_, O_i_, O_Zn_, or Zn_O_ point defect also is nonmagnetic. However, a strong spin splitting phenomenon is observed in the ZnO nanowire with V_Zn_ defect. The spin-up and spin-down DOSs are asymmetrical near the top of valence bands.

**Figure 5:**
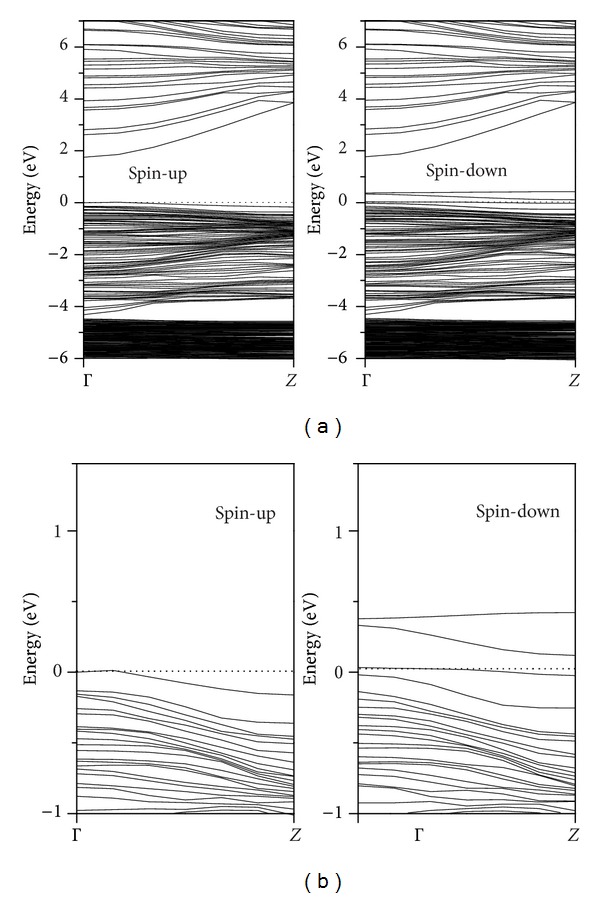
Calculated BS of ZnO nanowire with V_Zn_ defect. The corresponding BS near the Fermi level in Figure 5(a) is plotted in Figure 5(b).

By analysis of the BS of spin-up and spin-down electrons of the ZnO nanowire with V_Zn_ defect shown in Figure 5, it is found that the Fermi level moves from the spin-up to the spin-down band. The system shows half-metallic behavior in which the spin-up and spin-down electrons possess a Fermi surface, in agreement with the previous report [21]. In addition, as shown in Figure 5(b), three defect-related acceptor energy levels appear near the top of the spin-down valence bands. The upper two acceptor energy levels are deep and highly localized. The lower one is partially occupied by the electrons and is highly localized. This result reveals that the hole of the acceptor level cannot be excited easily into the valence bands, and the V_Zn_ defect has little contribution to the p-type electrical activity of ZnO. Our conclusion confirms the experiment results that V_Zn_ defects play minor role in p-type conductivity of ZnO [31].

**Figure 6:**
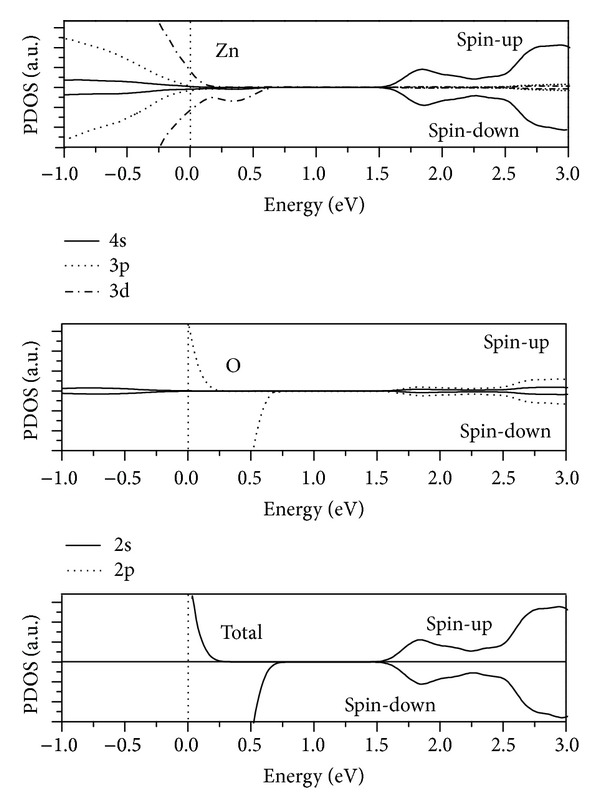
Calculated PDOS of ZnO nanowire with V_Zn_ defect.

By further analysis of the PDOS shown in Figure 6, it can be found that the metallic spin-down DOS near the Fermi level is mainly composed of O2p and Zn 3d states. In particular, O2p states do major contribution to the magnetic moment. This suggests that the appearance of the half-metallic ferromagnetism in ZnO nanorod with V_Zn_ originates from the hybridization of the O2p states with Zn 3d states.

Furthermore, the calculated Mulliken population analysis of the ZnO nanowire with V_Zn_ defect is listed in Table 2. Evidently, the O atoms around the V_Zn_ make major contribution to the observed magnetic moment. Magnetic moment of 0.562*μ*_B_, 0.556*μ*_B_ and 0.557*μ*_B_ is from each of the three O atoms in the basal plane and 0.102*μ*_B_ from the O atom in the axial plane. The residual magnetic moment is from the neighboring Zn atoms of the V_Zn_.

Structurally, at V_Zn_ in ZnO, there are four O atoms with dangling bonds pointing towards the vacancy site, where the dangling bonds feature O2p states. When this system is neutral, each dangling bond holds 1/2 electrons and interacts with each other to lower the total energy of the whole system. In this case, because of the tetrahedral structure of the four O atoms, the interaction between the dangling bonds results in hybrid orbital splitting. Therefore, the O atoms around the V_Zn_ make major contribution to the observed magnetic moment.

## 4. Conclusions

In conclusion, the effect of intrinsic point defect on the magnetic properties of ZnO nanowire is investigated by the first-principles calculation based on the DFT. The calculated BS, DOS, PDOS, and Mulliken population analysis results reveal that the pure ZnO nanowire without intrinsic point defect is nonmagnetic and ZnO nanowire with V_O_, Zn_i_, O_i_, O_Zn_, or Zn_O_ point defect also is nonmagnetic. However, a strong spin splitting phenomenon is observed in ZnO nanowire with V_Zn_ defect sitting on the surface site. At V_Zn_ in ZnO, there are four O atoms with dangling bonds pointing towards the vacancy site. Because of the tetrahedral structure of the four O atoms, the interaction between the dangling bonds results in hybrid orbital splitting.

## Figures and Tables

**Table 1:** Calculated relative energy Δ*ε* for the ZnO nanowire with different point defects (with the ground state energy taken as the reference value).

Defect	Δ*ε* (eV)
V_Zn1_	0.12
V_Zn2_	0.00
V_Zn3_	0.04
O_Zn1_	0.21
O_Zn2_	0.00
O_Zn3_	0.24
O_i1_	0.00
O_i2_	0.08
V_O1_	1.01
V_O2_	0.00
V_O3_	0.66
Zn_O1_	0.02
Zn_O2_	0.00
Zn_O3_	0.31
Zn_i1_	0.00
Zn_i2_	0.07

**Table 2:** Calculated Mulliken population of the ZnO nanowire with V_Zn_ defect. Around the V_Zn_, the O_ai_ represents the first-nearest neighboring O atoms in the basal plane, the O_c_ is the second-nearest neighboring O atoms in the axial plane, and the Zn_ai_ is the third-nearest neighboring Zn atoms in the basal plane.

Atom	*μ*_total_/*μ*_B_
O_a1_	0.562
O_a2_	0.556
O_a3_	0.557
O_c_	0.102
—	—
—	—
Zn_a1_	0.006
Zn_a2_	0.005
Zn_a3_	0.001
Zn_a4_	0.001
Zn_a5_	0.001
Zn_a6_	0.001
